# Cutaneous presentation of Double Hit Lymphoma: A Case Report With Review of the Literature

**DOI:** 10.1177/2324709616642592

**Published:** 2016-04-08

**Authors:** Yousef Khelfa, Yehuda Lebowicz

**Affiliations:** 1 Marshall University School of Medicine, Huntington, WV, USA

**Keywords:** double hit lymphoma, DLBCL, cutaneous lymphoma

## Abstract

Diffuse large B-cell lymphoma (DLBCL) is the most common type of non-Hodgkin lymphoma (NHL), representing approximately 25% of diagnosed NHL. DLBCL is heterogeneous disease both clinically and genetically. The 3 most common chromosomal translocations in DLBCL involve the oncogenes BCL2, BCL6, and MYC. Double hit (DH) DLBCL is an aggressive form in which MYC rearrangement is associated with either BCL2 or BCL6 rearrangement. Patients typically present with a rapidly growing mass, often with B symptoms. Extranodal disease is often present. Though there is a paucity of prospective trials in this subtype, double hit lymphoma (DHL) has been linked to very poor outcomes when patients are treated with standard R-CHOP. There is, therefore, a lack of consensus regarding the standard treatment for DHL. Several retrospective analyses have been conducted to help guide treatment of this disease. These suggest that DA EPOCH-R may be the most promising regimen and that achievement of complete resolution predicts better long-term outcomes.

## Case Presentation

**Figure 1.**
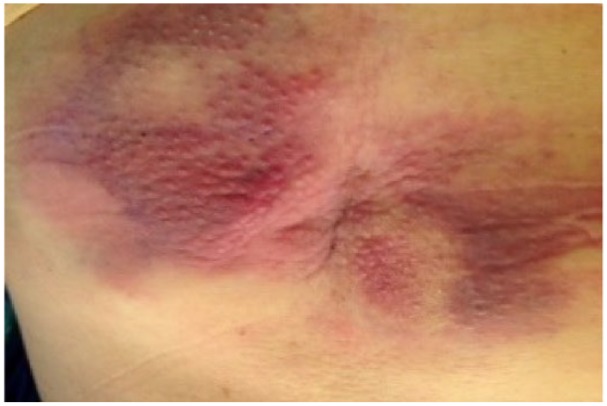
11 × 8 cm purple red skin nodule in mid-back on initial presentation.

**Figure 2.**
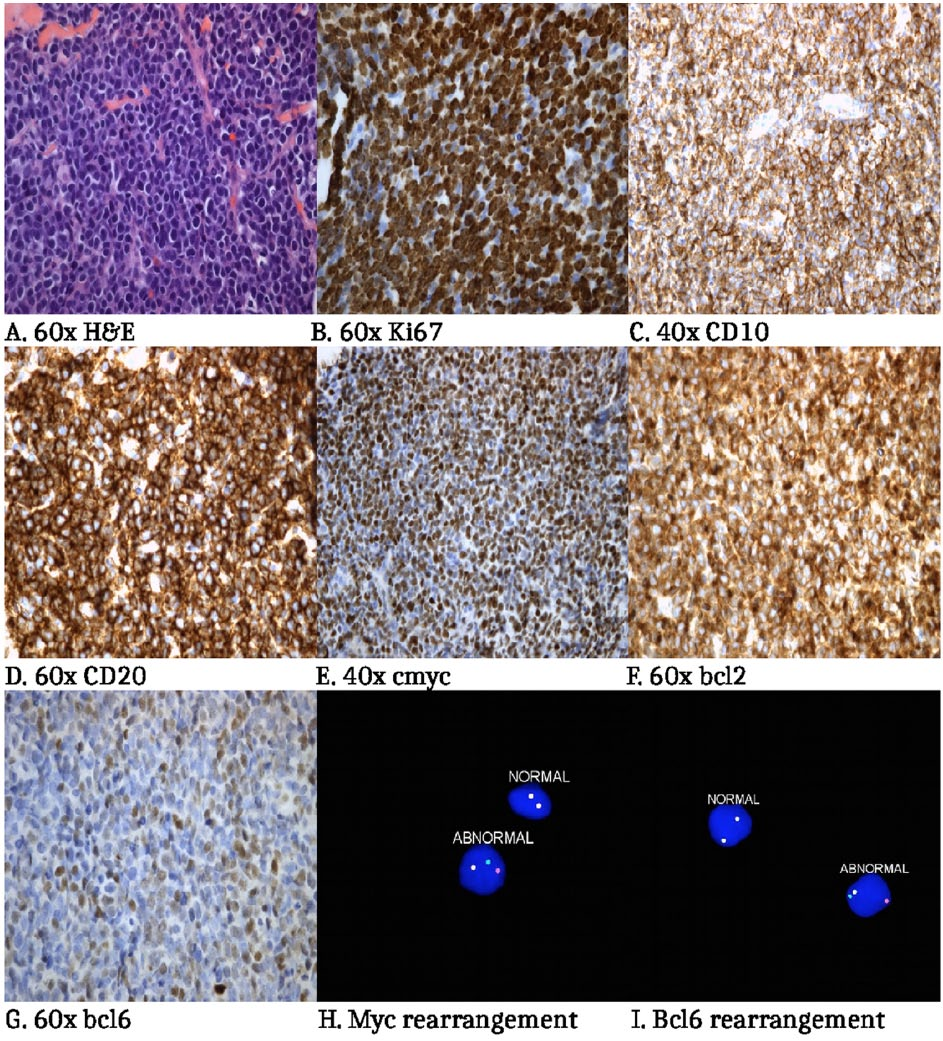
(A) A diffuse proliferation of medium-large mitotically active cells with irregular nuclear contours and nucleoli (60×, hematoxylin-eosin stain), (B) The tumor cells have a high proliferation index (Ki-67; 60×), (C) The tumor cells strongly express CD10 immunohistochemistry (40×), (D) CD20 immunohistochemistry (60×), (E) Myc (40×), (F) Bcl2 (60×), and (G) bcl6 (60×). FISH MYC (H) and BCL6 (I) rearrangements.

A 63-year-old Caucasian female with a history of diabetes mellitus had been complaining of lower mid-back cutaneous nodule that had been growing in size (Figure 1). The patient denied trauma, skin discharge, weight loss, fever, and night chills but had mild fatigue. A magnetic resonance imaging scan of lumbar spine was ordered by her primary doctor, which showed a diffuse abnormal signal over an area of about 11 cm × 8 cm. Ultrasound of the area showed an asymmetric abnormal echogenicity to the right of the midline. She was subsequently referred for a computed tomography–guided skin biopsy that demonstrated a diffuse infiltration of malignant cells of moderate size with ovoid to irregular nuclear contours and many nucleoli with significant mitotic activity. Flow cytometric analysis identified an abnormal population of cells accounting for the majority of events analyzed, showing surface lambda light chain restriction; coexpression of CD19, CD20, FMC-7, and HLA-DR; and aberrant coexpression of both CD10 and CD5. Immunohistochemical stains for CD20, CD10, BCL2, MUM1, and c-MYC were strongly positive, BCL6 was moderately positive, and the malignant cells had a proliferation index (Ki-67) of greater than 90%. CD3 and Cyclin D1 were negative (Figure 2A-G). Fluorescence in situ hybridization (FISH) analysis was performed using DNA probes for aggressive B-cell lymphoma. Two hundred interphase nuclei were examined for each probe and revealed a rearrangement of the MYC gene in 93.5%, a rearrangement of the BCL6 gene in 97.5%, and a gain of BCL2/18q21 in 69.5% of nuclei. There was no evidence of a BCL2 gene rearrangement (Figure 2H and I).

## Introduction

Diffuse large B-cell lymphoma (DLBCL) is a heterogeneous disease both in molecular pathogenesis and clinical outcome. Double hit (DH) DLBCL is an aggressive subtype of DLBCL in which there is both a MYC rearrangement as well as either a BCL2 or less commonly a BCL6 rearrangement. When all 3 are present it may be referred to as a triple hit lymphoma (THL).^1^ Though immunohistochemistry (IHC) can be used to detect the over expression of MYC, BCL2, and BCL6 protein, these are not necessarily diagnostic of double hit lymphoma (DHL). Rather, when it is present by IHC in the absence of their rearrangement, detected by FISH, it can be classified as another entity of DLBCL called dual expresser (DE).^2^ The presence of MYC and BCL2 rearrangement is common in DH DLBCL, while rearrangement of BCL6 is rare.^3^ DH DLBCL is typically of germinal center B-cell origin, commonly referred to as GCB; DE DLBCL is typically of non–germinal center origin, non-GCB. Both are often aggressive and may not be adequately treated with standard R-CHOP therapy. Some of the confusion lies in recent discoveries, rapidly changing landscape leading to new and still evolving terminology. Thus, the World Health Organization does not yet list these as separate/distinct subtypes. Rather, they all fall under the general heading of DLBCL NOS, or B-cell unclassifiable between DLBCL and Burkitt lymphoma.^4^ The anxiously anticipated update to the 2008 classification is expected in the near future and will likely reflect much of these changes.

## Clinical Presentation: When to Test for DHL

In a large retrospective Japanese study, Niitsu et al found that extranodal sites, B symptoms, advanced stage, high serum lactate dehydrogenase (LDH) level, and bone marrow involvement were significantly more prevalent among patients with the dual translocation. Among the 19 patients with the dual translocation, extranodal sites of disease were bone marrow (16 patients), pleural effusion (6 patients), central nervous system (4 patients), small intestine (2 patients), stomach (2 patients), lung (1 patient), thyroid (1 patient), and breast (1 patient). In addition, 12 patients had at least 2 extranodal localizations.^5^

On the other hand, in a smaller series of 53 patients published by Landsburg et al, DH gene rearrangements were detected in 32% of patients. In their series, no baseline factor, including age, stage, LDH, International Prognostic Index (IPI) score, or histology were statistically significant in association with DH status.^6^

There is no clear consensus regarding who and when to test for DHL status. Both **NCCN** (National Comprehensive Cancer Network) and ESMO (European Society for Medical Oncology) suggest but do not require evaluation in their most recent guidelines.^7,8^

## DHL and Genetic Abnormalities

With the introduction of gene expression profiling in the last decade or so, DLBCL has been found to actually be a heterogeneous group of lymphoid tumors that are diverse not only in their underlying molecular pathogenesis but also in their clinical behavior and outcome. DLBCL has since been classified under 2 main groups, the germinal center type (GC) and the activated B-cell type (ABC).^9^ The first group (GC) resembles a normal germinal center B cell and has a superior rate of 5-year survival with current therapy (CHOP or R-CHOP), whereas the second group resembles an activated B cell and is associated with inferior rates of 5-year survival. As gene expression profiling is not yet a commercially available tool, clinicians often rely on HANS algorithm using available IHC stains to differentiate the germinal center and non–germinal center types.^10^

The 3 most common chromosomal translocations in DLBCL involve the following oncogenes: (*a*) BCL2 (gene rearrangement at 18q21), found in more than 30% DLBCL, mainly in the GC molecular subtype, which when present as the only abnormality does not affect DLBCL survival outcome^11^; (*b*) BCL6 (gene rearrangement at 3q27) seen in about a third of patients with no prognostic value when present alone^12^; (*c*) and c-MYC (gene rearrangement at 8q24), seen in up to 15% of cases of DLBCL, which alone portends a worse prognosis following treatment with standard doxorubicin-based combination chemotherapy.^13^

## DHL, Dual Expresser Lymphoma (DEL), and Triple Hit Lymphoma (THL)

Friedberg et al^14^ stated that the combination of genetic abnormalities c-MYC and often BCL2 rearrangements, also called DH DLBCL, has been established as predicting for highly refractory disease after conventional therapy (eg, R-CHOP). Less frequently DH DLBCL may have translocations involving c-MYC and BCL6, and more rarely what studies in past decade called “triple-hit” lymphoma when the c-MYC, BCL2, and BCL6 rearrangements present concordantly; most of the studies analyzed data from patients with THL indicate inferior survival when treated with the conventional treatment.^15^

The expression of BCL2 protein is not always correlated with the t(14;18); Iqbal et al^16^ found that BCL2 protein expression was observed in 44% of GCB DLBCL and 62% of ABC DLBCL, and was correlated with a poor outcome with R-CHOP standard therapy only in the GCB subtype and not in the ABC subtype. In the same study, the ABC subtype of DLBCL rarely had the t(14;18), yet amplifications of 18q21 were seen in up to two thirds of cases, providing a possible mechanism for BCL2 overexpression in these tumors.

## Optimal Management of DHL

Due to its rarity and resultant paucity of prospective trials in DHL, there is no consensus about the standard treatment for DHL or DEL. Multiple retrospective analyses were conducted to help guide the treatment of this aggressive and heterogeneous form of DLBCL (Table 1).

**Table 1.** A Summary of Multiple Retrospective Analyses Conducted to Help Guide the Treatment of DHL.

Study	Year Published	DHL N/%of Total Size	Study Type	Treatment	Median Age	Survival Outcome
Johnson et al^13^	2009	54; DLBCL or BCLU in 98%	Retrospective analysis	CHOP ± R; (63%); HD chemo; Other	52% were >60 years of age	Median OS 1.4 years and 1 year in R-CHOP and CHOP
Dunleavy et al^17^	2011	66; 20% with high MYC/BCL2	Retrospective analysis from a prospective study	EPOCH-R	48 years	10-year survival compared in 4 groups: MYC^+^/BCL2^+^ vs all others (MYC^+^/BCL2^−^, MYC^−^BCL2^+^, MYC^−^/BCL2^−^). Global *P* = .5 (PFS) and *P* = .8 (OS)
						R-EPOCH overcome inferiority of DHL
Petrich et al^18^	2014	311 (100%)	Multicenter retrospective analysis	DA EPOCH-R 64 (21%)	60 years	mFollow-up 23 months
				R-HYPERCVAD 65 (21%)		mPFS 10.9 months, mOS 21.9 months
				R-CODOX-MIVAC 42 (14%)		SCT after CR/all regimen on OS benefit
				R-CHOP 100 (32%)		Better mPFS 26.6 months, all intensive regimens vs R-CHOP 7.8 months
Snuderl et al^19^	2010	20 (100%)	Single institution retrospective analysis	R-ICE + MTX/ASCT (1); CHOP (1); R-CHOP (3); R-CHOP + MTX (6); R-CHOP + MTX ASCT (1); R-EPOCH + MTX (3); CODOX- + MTX/R-IVAC (3); P (1); NK(1)	64 years	ORR 10/20** (50)
						mOS 0.38 year
Li et al^20^	2012	52; DLBCL or BCLU in >90%	Retrospective analysis	R-CHOP or R-Hyper-CVAD	55 years	Median OS of 18.6 months; more intense therapy (*P* = .54) or SCT (*P* = .73) was not associated with a better outcome
Oki et al^21^	2014	129 (72% MYC/BCL2)	Single institution retrospective analysis	R-EPOCH	62 years	Overall 2-year EFS 33%
				R-HYPERCVAD/MA		Better OS R-EPOCH vs R-CHOP (*P* value of .057)
				R-CHOP		SCT did not improve OS
						CR R-EPOCH (68%), R-HYPERCVAD (70%), R-CHOP (20%)
**Niitsu** et al^5^	2009	19 (100%)	Retrospective analysis from a prospective study	CyclOBEAP (6); CHOP + HD MTX (3); CHOP (4); R-CHOP (3), CyclOBEAP + R (3)	61 years	ORR 17/19 (89%)
						mOS 1.5 year
Tomita et al^22^	2009	27 (100%)	Retrospective analysis	CHOP or CODOX-M/IVAC or HyperCVAD (+R, n =14; −R, n = 8)	51 years	ORR 6/23 (26%)
						mOS 0.5 year
Gandhi et al^23^	2013	106/DLBCL or BCLU in >95%	Retrospective analysis	R-CHOP; DA-EPOCH-R; R-Hyper-CVAD; CODOX-M/IVAC	60 years	Median PFS and OS of 9 and 12 months; DA-EPOCH-R resulted in superior CR compared with R-CHOP (*P* = .01) and other intensive regimens (*P* = .07); lower rate of primary refractory disease with DA-EPOCH-R compared with R-CHOP (*P* = .005); no improvement in OS in CR
Le Gouill et al^24^	2007	16 (100%)	Retrospective analysis	CEEP/COPADM + Auto-SCT/BEAM (1); CHOP/IVAM (1); COPADM/CYVE (3); COPADM (1); COPADM + Auto-SCT/BEAM (1); COPADM + Allo-SCT/Bu/Cy (1); CEEP/DHAP + Auto-SCT/BEAM (1); R-CHOP (4); CHOP (1); Steroids# (1); R-CEEP Allo-SCT/TBI/Cy (1)	61 years	ORR 12/16 (75%)
						mOS 0.42 years
Kanungo et al^25^	2006	14 (100%)	Retrospective analysis	CT-NOS (11); R (1); CT and BMT (1); CT, BMT, and RT (1)	55 years	<1 year
Dunleavy et al^26^	2015	52 (45%)	Prospective analysis of MYC-rearranged aggressive B-cell lymphoma	R-DA-EPOCH	61 years	14-month OS 79%
						14-month PFS 86%

Abbreviations: CR, complete resolution; DHL, double hit lymphoma; DLBCL, diffuse large B-cell lymphoma; OS, overall survival; PFS, progression-free survival; SCT, stem cell transplantation; ORR, overall response rate; BCLU, B-cell lymphoma unclassifiable (with features intermediate between DLBCL and Burkitt lymphoma).

**Figure 3.**
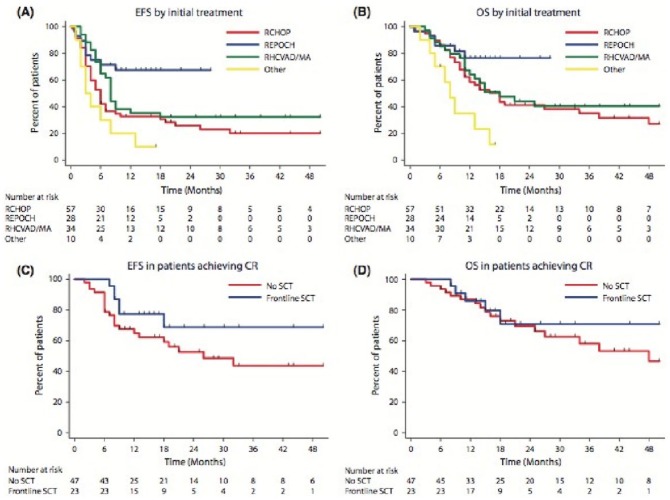
Survival by treatment. (A) Event-free survival by initial treatment. (B) Overall survival by initial treatment. (C) Event-free survival in patients who achieved CR, based on whether frontline stem cell transplant was performed. (D) Overall survival in patients who achieved CR, based on whether frontline stem cell transplant was performed. EFS, event-free survival; OS, overall survival; R-CHOP, rituximab, cyclophosphamide, doxorubicin, vincristine, prednisone; REPOCH, rituximab, etoposide, prednisone, vincristine, cyclophosphamide, doxorubicin; RHCVAD/MA, rituximab, hyperfractionated cyclophosphamide, vincristine, doxorubicin, dexamethasone, alternating with cytarabine plus methotrexate; SCT, stem cell transplantation. (Reproduced with permission of John Wiley and Sons.)

Petrich et al^18^ published a multicenter retrospective analysis looking at DH DLBCL patients who were treated, and compared the outcomes of those that received standard R-CHOP versus more intensive regimens (R-HYPERCVAD, DA EPOCH-R, or R-CODOX-MIVAC). Also, they looked at patients who had complete resolution (CR) and underwent stem cell transplantation (SCT) and whether that improved their overall survival (OS). After a median follow-up of 23 months, the median progression-free survival (PFS) and median OS for all patients were 10.9 months and 21.9 months, respectively, with no difference in OS for those that received intensive regimens or had SCT after CR over those who got R-CHOP or those who did not have SCT. However, median PFS was significantly better for intensive regimen patients over R-CHOP patients, 26.6 months versus 7.8 months, with a *P* value of .0463 for the DA EPOCH-R group, .001 for the R-HYPERCVAD group, and .036 for the R-CODOX/M IVAC group. Of note, there was no difference between the 3 intensive treatment regimens. In another study, Oki et al^21^ analyzed the outcome of 129 cases of DHL at MD Anderson; DHL was defined as B-cell lymphoma with translocations and/or extra signals involving MYC plus BCL2 and/or BCL6. The 2-year event-free survival (EFS) rate in all patients was 33%; however, when analyzed by individual regimen, those who received R-CHOP, R-EPOCH, and R-HYPERCVAD/MA had 2-year EFS of 25%, 67%, and 32%, respectively. Autologous SCT after CR did not improve OS in patients achieving complete response with initial therapy (n = 71). In addition, 2-year EFS rates in patients who did (n = 23) or did not (n = 48) receive frontline SCT were 68% and 53%, respectively (*P* = .155; Figure 3).

A recent prospective multicenter phase II study using R-DA-EPOCH for MYC rearranged aggressive B-cell lymphoma was presented by Dunleavy at ASH, which demonstrated a short-term median follow-up time of 14 months, PFS, time to progression (TTP), and OS were 79%, 86%, and 77%, respectively, for all patients.^26^

The most consistent result of these retrospective analyses indicates that DHL has an inferior outcome when treated with standard R-CHOP therapy. Furthermore, they imply that this inferior outcome may be overcome by using more intense regimens such as R-DA-EPOCH, R-HYPERCVAD, or R CODOX/M IVAC. Based on the MD Anderson experience, R-DA-EPOCH may be the best tolerated while maintaining the largest improvement in PFS.

Until a prospective trial demonstrates improved survival, these suggestions will remain somewhat speculative. Table 1 summarizes the largest series.

Several prospective trials are ongoing that will hopefully answer some of these questions (Table 2). A number of these trials are looking at multiple targets involved in the pathogenesis of lymphoma at the molecular and genetic levels.

**Table 2.** Ongoing Prospective Trials on Targeted Therapy for B-cell Lymphoma.

ID Number	Title
NCT02272686	Targeting BTK With Ibrutinib After Autologous Stem Cell Transplantation in “Double-Hit” B-Cell Lymphoma
NCT02213913	Prospective, Multi-center Phase I/II Trial of Lenalidomide and Dose-Adjusted EPOCH-R in MYC-Associated B-Cell Lymphomas
NCT01856192	Randomized Phase II Open Label Study of Lenalidomide R-CHOP (R2CHOP) vs RCHOP (Rituximab, Cyclophosphamide, Doxorubicin, Vincristine and Prednisone) in Patients With Newly Diagnosed Diffuse Large B Cell Lymphoma
NCT01092182	Phase II Study of Dose-Adjusted EPOCH+/-Rituximab in Adults With Untreated Burkitt Lymphoma, c-MYC Positive Diffuse Large B-Cell Lymphoma and Plasmablastic Lymphoma
NCT02110563	Phase I, Multicenter, Cohort Dose Escalation Trial to Determine the Safety, Tolerance, and Maximum Tolerated Dose of DCR-MYC, a Lipid Nanoparticle (LNP)-Formulated Small Inhibitory RNA (siRNA) Oligonucleotide Targeting MYC, in Patients With Refractory Locally Advanced or Metastatic Solid Tumor Malignancies, Multiple Myeloma, or Lymphoma
NCT01949883	A Phase 1 Study of CPI-0610, a Small Molecule Inhibitor of BET (Bromodomain and Extra-terminal) Proteins, in Patients With Progressive Lymphoma
NCT01181271	Sequential Myeloablative Autologous Stem Cell Transplantation Followed by Allogeneic Non-Myeloablative Stem Cell Transplantation for Patients With Poor Risk Lymphomas
NCT02226965	A Phase II Study of PNT2258 in Patients With Relapsed or Refractory Diffuse Large B-Cell Lymphoma
NCT01897012	A Phase 1 Trial of MLN8237 Plus Romidepsin for Relapsed/Refractory Aggressive B-Cell and T-Cell Lymphomas
NCT01490723	Dose-Intense Yttrium-90 Ibritumomab Tiuxetan (Zevalin)-Containing Non-Myeloablative Conditioning for Allogeneic Stem Cell Transplantation in B-cell Malignancies

The Ohio State group, in a recently published article in *Cancer* (median of 28.5 months follow-up) reviewed the outcome of their treated patients with MYC+ and DH, and they demonstrated that only age and achievement of CR was correlated with better outcomes. The median PFS for patients with documented double-hit NHL who achieved a CR had not yet been reached (95% confidence interval [CI] = NR to NR) versus 3.9 months (95% CI = 1.8–8.0 months) for those who did not achieve a CR (*P* < .0001). The median OS for those patients who did not achieve a physician-assessed CR was 7.0 months (95% CI = 2.0-12.5 months) compared with a median not reached for those who did achieve a CR (95% CI = NR to NR; *P* < .00001).^27^

## Conclusion and Management of Our Patient

**Figure 4.**
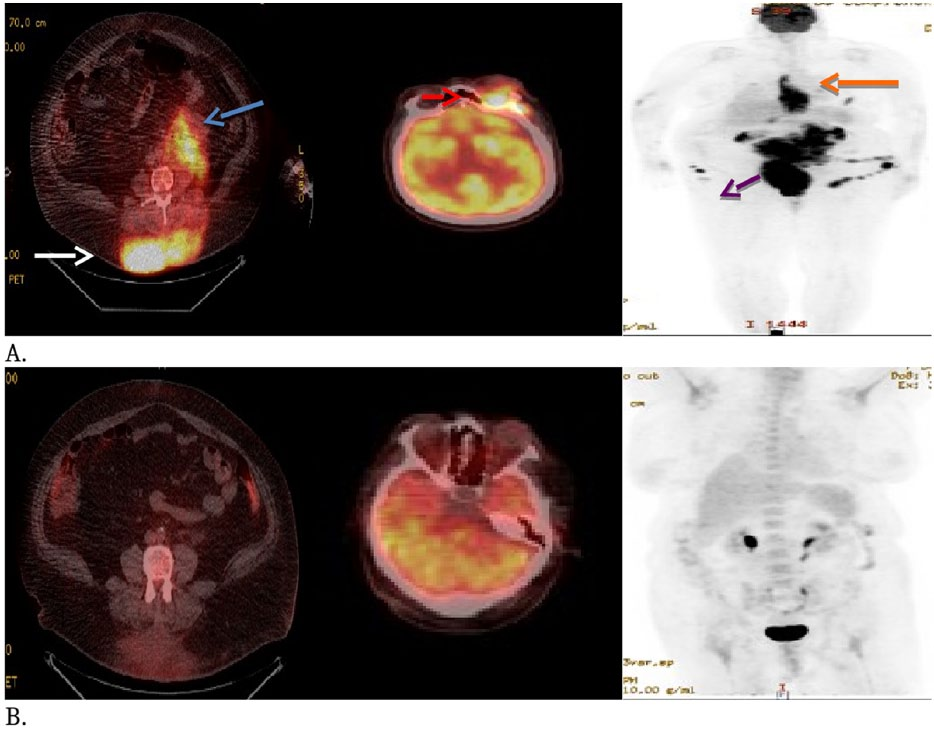
PET/CT. (A) Hypermetabolic orbital mass (red arrow), posterior mediastinal mass (orange arrow), retroperitoneal mass (blue arrow), mass within the midline lumbar subcutaneous tissues (white arrow), diffuse hypermetabolic activity between the bladder and rectum that corresponds to the uterus (purple arrow). (B) Complete resolution of hypermetabolic soft tissue activity.

**Figure 5.**
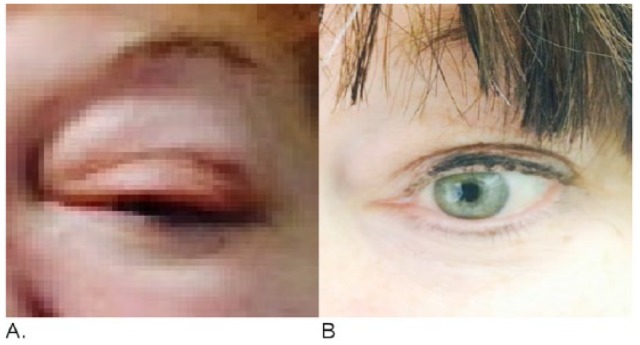
Left orbital swelling (A) has completely resolved (B).

**Figure 6.**
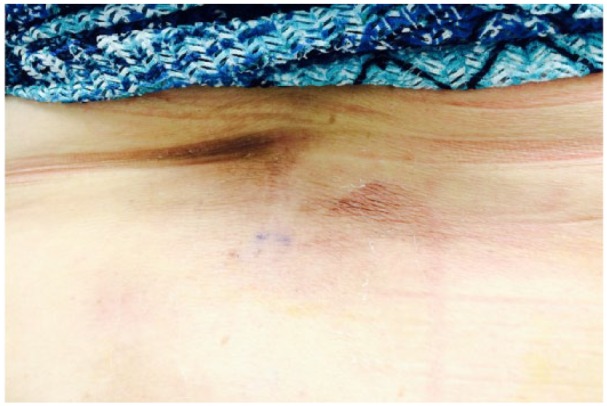
Significant improvement in patient’s mid-back skin nodule size and discoloration after second cycle of DA-EPOCH-R.

Back to our 63-year-old patient who was diagnosed with not only rare extranodal presentation of her DH DLBCL but also with a rare coexpression between MYC and BCL6 rearrangement. Her physical exam was consistent with her back skin nodule described above (Figure 1) with left eye protrusion and swelling but no lymphadenopathy, and the rest of her exam was normal. We completed her staging with positron emission tomography/computed tomography (PET/CT), bone marrow biopsy, lumbar puncture, as well as checking comprehensive metabolic panel, complete blood count, LDH, uric acid, HIV, viral hepatitis panel, and 2D echo. All tests came back within normal range except, LDH of 503 and abnormal PET/CT scan as shown in Figure 4A). Patient has stage IVE DH DLBCL, with IPI score 4.^28^ Reviewing the literature as above, R-DA-EPOCH was the treatment option we favored, outside of a clinical trial. The patient’s excellent functional status helped making this decision easy for both us and the patient. After 2 cycles of R-DA-EPOCH, she had complete resolution of her left eye protrusion and swelling (Figure 5), near complete resolution of her mid-back skin nodule (Figure 6), and significant improvement of her disease as repeated PET scan showed (Figure 4B) with no areas displaying FDG avidity.

The patient also received central nervous system prophylaxis, with IT Methotrexate. She completed 6 cycles, without significant toxicity except for 1 episode of neutropenic fever requiring admission, short hospitalization, and dose reduction in therapy. Repeat PET/CT posttreatment continues to demonstrate CR. As shown by The Ohio State group, CR should correlate with improved long-term outcomes. After 4 months she developed lower back pain and paresis of left lower extremity. Lumbar puncture showed white blood cell count of 3000. Cytology and flow cytometry confirmed leptomeningeal lymphomatosis.

She was started on HDMTX and her white blood cell count decreased to 100; however, pain and paresis persisted. Her quality of life was severely affected and she chose to pursue comfort measures.
